# Protocol optimization to process mouse colon samples for single-nuclei RNA sequencing using FLEX library preparation

**DOI:** 10.1016/j.xpro.2026.104581

**Published:** 2026-05-15

**Authors:** Jin-Hee Kim, Annalyssa N. Long, Yumo Xie, Kelly T. Carter, Sophia R. Noehl-Tekorius, Anna E. Elz, Ming Yu

**Affiliations:** 1 Translational Science and Therapeutics Division, Fred Hutchinson Cancer Center, Seattle, WA 98109, USA; 2 Immunotherapy Integrated Research Center, Fred Hutchinson Cancer Center, Seattle, WA 98109, USA

**Keywords:** Single Cell, Genomics, RNAseq, Gene Expression

## Abstract

Single-nuclei RNA sequencing enables the interrogation of tissue microenvironments in clinical samples preserved by snap-freezing, where cell membrane damage precludes single-cell isolation, but nuclei remain intact for high-quality molecular profiling. Here, we present an optimized protocol to process mouse colon samples for single-nuclei RNA sequencing using FLEX library preparation. We describe steps for preparing and dissociating colon tissue, nuclei filtration, and fixation. We then detail procedures for Chromium Fixed RNA Profiling (Gene Expression Flex).

## Highlights

• Instructions for the isolation of intact nuclei from flash-frozen mouse colon tissue
• Steps for nuclei filtration and fixation for probe hybridization and FLEX prep
• Guidance on quality control for downstream single-nuclei RNA sequencing

## Abstract (editor highlights)

Publisher’s note: Undertaking any experimental protocol requires adherence to local institutional guidelines for laboratory safety and ethics.

## Graphical abstract

**Figure:**
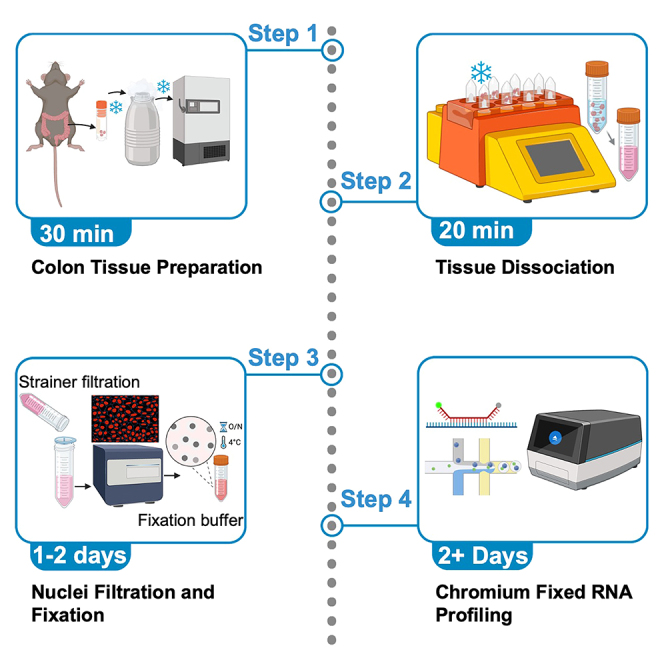

## Before you begin

This protocol outlines the workflow for isolating mouse colon tissue, snap-freezing samples, and generating high-quality, single-nuclei suspensions for downstream library preparation. The method is optimized for 30–70 mg of flash frozen mouse colon tissue, yielding comparable nuclei recovery. This method has been validated across a wide range of mouse ages (3 to 23 months) and in both sexes, and no major differences in nuclei recovery or quality have been observed. The workflow includes procedures for mouse handling, CO_2_-based euthanasia, and the use of gentleMACs™ Octo Dissociator with coolers, which together support efficient and reproducible nuclei isolation.

### Innovation

This protocol introduces an optimized workflow that enhances the 10× Genomics Fixation of Cells & Nuclei for Chromium Fixed RNA Profiling (Gene Expression Flex) by integrating key elements of the Miltenyi nuclei isolation procedure. Single-nuclei RNA sequencing avoids dissociation-driven gene activation,^1^^,^^2^ providing a more accurate snapshot of true cellular gene expression and is well suited for snap-frozen tissue. The combination of these two methodologies enables the generation of high quality, structurally intact nuclei from challenging tissues while fully preserving compatibility with the 10× Genomics Fixation chemistry and downstream library construction.

### Institutional permissions

All animal procedures were reviewed and approved by the Institutional Animal Care and Use Committee (IACUC) of Fred Hutch Cancer Center prior to the start of the study under protocol number 1624.

### Preparation one

#### Obtain colon tissue

**Timing: 10 min**

**Figure 1:**
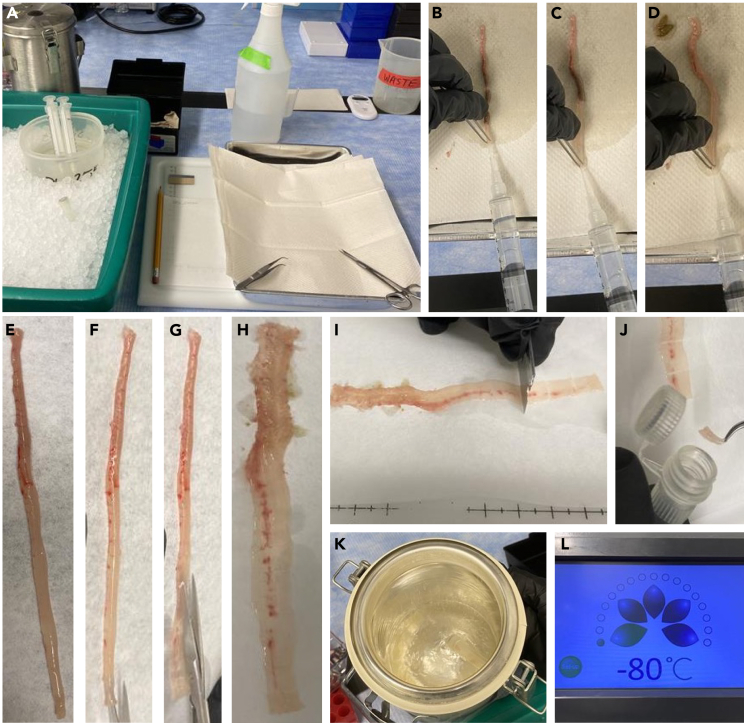
Colon tissue collection and preparation for single nuclei isolation (A) Bench setup for mouse colon dissection. (B–D) Flushing of colon lumen with ice-cold PBS using syringe to remove luminal contents. (E–H) Placement of colon on bibulous paper followed by longitudinal openings to expose the mucosal surface. (I) Isolation of an approximately 1 cm segment form the designated colon region using a razor blade. (J) Transfer of a tissue section into a cryotube. (K) Rapid freezing of the sample by immersion of the cryotube in liquid nitrogen. (L) Storage of frozen samples at −80°C until further processing.

***Note:*** Each isolation procedure requires 1 dissection tray, 1 tweezer, 1 surgical scissor, 20 ml of sterile-filtered 1× DPBS, 1 bibulous paper, 10 ml syringe, 1 razor blade, 1 cryotube, liquid nitrogen, −80°C deep freezer. While RNase-free DPBS is recommended, it is not strictly required at this stage. All materials, including tweezers and scissors, should be thoroughly cleaned using RNase decontamination solutions (e.g., RNase*Zap*™) before use.

1. Prepare reagents and workstation (Figure 1A).
  a. Fill a benchtop liquid nitrogen (LN_2_) container with LN_2_.
  b. Place 1× DPBS on ice to chill.
  c. Prepare CO_2_ euthanasia station according to approved institutional animal care and use committee (IACUC) protocols.

### Preparation two

#### Isolate nuclei from snap-frozen colon tissue

**Timing: 20 min**

2. Ensure that the nuclei dissociation program 4°C_nuclei_1 is programmed on the gentleMACS™ Octo Dissociator system. If the 4°C_nuclei_1 program is not on your dissociator, contact your Miltenyi representative for help uploading the program.
3. Prechill nuclei isolation equipment.
  a. Place Octo Coolers in the −20°C freezer the day before the experiment.
  b. Cool centrifuge to 4°C before use.
4. Prechill nuclei isolation consumables.
  a. Place all plastic consumables that will come into contact with the nuclei in the refrigerator for at least 1 h, or overnight prior to beginning the experiment.
5. Prepare 4 mL of lysis buffer per sample and place on ice.
6. Prepare resuspension buffer.
7. Prepare 0.5 mL of fixation buffer B per sample, following 10× Genomics Document CG000782.

***Note:*** Each nuclei isolation requires the following: 1 Octomacs C tube, 1 Octo Cooler, 1 15 mL conical tube, nuclei extraction buffer, RNase inhibitor, 1 70 μm MACS SmartStrainer, 1 30 μm MACS SmartStrainer, 0.5mL of fixation buffer B.

**CRITICAL:** Adding an RNase inhibitor to the nuclei extraction buffer is essential for preserving RNA integrity. Strictly adhere to the specified lysis buffer components.

## Key resources table

**Table:** 

REAGENT or RESOURCE	SOURCE	IDENTIFIER
**Chemicals, peptides, and recombinant proteins**		

Nuclease Free Water	Cytiva	SH30538.03
RNase inhibitor	10× Genomics	1000887
10% BSA	Sigma Aldrich	126615
Nuclei extraction buffer	Miltenyi	130-128-024
Acridine Orange	Revvity	CS1-0108
37% Formaldehyde	Fisher Bioreagents	BP531-500
DPBS (1×)	Gibco	14190-144
Bibulous Paper	Fisher Scientific	12-587-112
RNase*Zap*	Invitrogen	AM9780
Glycerol (Molecular Biology)	Fisher Scientific	BP229-1
Trypan blue 0.4%	Bio-rad	1450021
30 μm pre separation filter	Miltenyi	130-041-407
1.5 ml DNA LoBind Tube	Eppendorf	022431021
5 ml DNA LoBind Tube	Eppendorf	0030122348

**Critical commercial assays**		

GEM-X Flex gene expression mouse 4-plex, 16 samples -GEM-X Flex GEM & Library Kit, 4 rxns (Reducing Agent B /Amp Mix C/Pre-Amp Primers B/GEM Enzyme Mix B/GEM Reagent Mix) -GEM-X Flex Gel Bead Kit, 4 rxns -GEM-X Flex Hybridization & Wash Kit, 24 rxns (Hyb Buffer B/Conc. Post-Hyb Buffer B/Enhancer) -GEM-X Flex Mouse Transcriptome Probe Kit, 16 samples	10× Genomics	1000797
GEM-X Flex sample preparation v2 kit -Conc. Fix & Perm Buffer B -Conc. Quench Buffer B -Additive C -Enhancer	10× Genomics	1000781
GEM-X Flex gene expression chip kit -Partitioning Oil B -Recovery Agent -FX Chip & Gaskets	10× Genomics	1000791
Dual index TS Set A	10× Genomics	1000251
High Sensitivity D1000 ScreenTape	Agilent	5067-5584
High Sensitivity D1000 Sample Buffer	Agilent	5067-5603
High Sensitivity D1000 ladder	Agilent	5067-5587

**Software and algorithms**		

SoupX	Young et al.^3^	N/A
DoubletFinder	McGinnis et al.^4^	N/A
Seurat v5	Hao et al.^5^	
Cell Ranger	10× Genomics	N/A

**Other**		

gentleMACS C tube	Miltenyi	130-093-237
gentleMACS Octo Coolers	Miltenyi	130-130-533
Cellaca MX	Revvity	N/A
Chromium X	10× Genomics	N/A
C1000 touch thermal cycler	Bio-rad	N/A
TapeStation	Agilent	N/A
gentleMACS Octo Dissociator	Miltenyi	130-134-029
MACS SmartStrainers (30 μm)	Milteny	130-098-458
MACS SmartStrainers (70 μm)	Miltenyi	130-098-462

**Experimental models: Organisms/strains**		

*Mus musculus*	National Institute on Aging	C57BL/6JN

## Materials and equipment

**Table:** Lysis Buffer

Reagent	Final concentration	Amount for 1 sample
Nuclei extraction Buffer	NA	3.98 mL
RNase inhibitor (40 units/μL)	0.2 units/μL	20 μL

Make shortly before use. Maintain on ice.

**Table:** Nuclei Resuspension Buffer

Reagent	Final concentration	Amount for ∼1ml
Nuclei extraction Buffer	14%	140 μL
10% BSA	0.04%	4 μL
1× PBS	N/A	860 μL
RNase inhibitor (40 units/μL)	0.2 units/μL	5 μL

Make reagent shortly before use. Maintain resuspension buffer on ice.

**Table:** Fixation Buffer B

Reagent	Final concentration	Amount for 1 sample
Nuclease Free Water	NA	435 μL
Conc. Fix and Perm Buffer B (10×)	1×	55 μL
Formaldehyde (37%)	4%	60 μL

Make reagent shortly before use. Maintain at 20°C**–**24°C.

**Table:** Quenching Buffer B

Reagent	Final concentration	Amount for 1 sample
Nuclease Free Water	NA	962.5 μL
Conc. Quench Buffer B	1×	137.5 μL

Make reagent shortly before use. Maintain on ice.

**Table:** Post-Hyb Wash Buffer B

Reagent	Final concentration	Pooling 4 samples
Nuclease Free Water	NA	4.95 mL
Conc. Post-Hyb Buffer B	1×	0.275 mL
Enhancer	1×	0.275 mL

Prepare reagent shortly before use and maintain it at 20°C**–**24°C. Prepare the enhancer by heating it at 65°C for 10 min, then vortex and add it to the other solution.

**Table:** Post-Hyb Resuspension Buffer B

Reagent	Final concentration	Amount for 1 pool
Nuclease-free Water	NA	783.75 μL
Conc. Post-Hyb Buffer B	NA	41.25 μL

Prepare reagent shortly before use. Pipette mix and maintain on ice.

**Table:** GEM Master Mix

Reagent	Final concentration	Amount for 1 GEM Reaction
GEM Reagent Mix	NA	19.9 μL
Reducing Agent B	NA	1.6 μL
GEM Enzyme B	NA	11.8 μL

Prepare reagent shortly before use. Pipette mix and maintain on ice.

## Step-by-step method details

### Mouse colon isolation

**Timing: 15 min**

1. Euthanize the mouse with CO_2_ chamber followed by cervical dislocation.
2. Make vertical midline incision and identify the colon.
3. Cut the distal colon and carefully pull it outward toward the cecum to isolate intact colon.
4. Load a 10 mL syringe with ice-cold PBS and flush the colon lumen repeatedly until the outflow appears clear (Figures 1B–1D).

### Snap-freeze a colon segment

**Timing: 10 min**

5. Place colon on bibulous paper (Figure 1E).
6. Open the colon longitudinally to expose the mucosal surface (Figures 1F–1H).
7. With a razor blade, isolate a ∼1 cm section from designated area of colon and place it directly into a cryotube (Figures 1I and 1J).
8. Place the cryotube into LN_2_ to rapidly freeze the sample (Figure 1K).
9. Store the sample at −80°C deep freezer (Figure 1L).

***Note:*** Tissue can be stored at −80°C for up to 12 months. For longer-term storage, keep the tissue in a LN_2_ tank.

### Isolate nuclei from snap-frozen colon tissue

**Timing: 30 min**

***Note:*** For the remaining steps, use pipette tips and serological pipettes with filters to reduce the chance of contamination and RNA degradation. We have also found that processing up to 4 samples at a time for nuclei isolation and fixation is most manageable and reduces the time between nuclei isolation and getting them into fixation.

**Figure 2:**
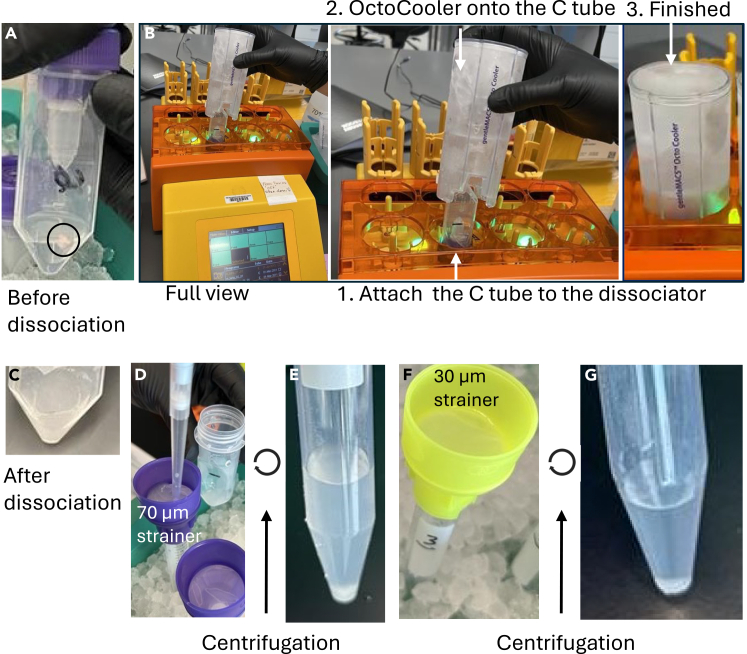
Nuclei isolation from snap-frozen colon tissue (A) Snap-frozen colon tissue is transferred into a C tube containing nuclei lysis buffer. (B) Tissue is dissociated using the gentleMACS Dissociator equipped with Octo Cooler. Left to right: full view of the dissociator -> assembly of the C tube and OctoCooler -> completed assembly. (C) Representative image of tissue following mechanical dissociation. (D) The homogenate is passed through 70 μm filter to remove debris. (E) Filtered homogenate is centrifuged to pellet nuclei. (F) The supernatant is removed, and nuclei pellet is resuspended, passed through 30 μm filter, and centrifuged to obtain purified nuclei preparation (G).

***Note:*** Weigh the snap-frozen tissue to make ensure it is within 30–70 mg range, as this protocol is optimized for that amount. Keep the tissue in the cryotube on dry ice at all times and weigh it quickly after tare the balance with an empty cryotube.

10. Add 2 mL of lysis buffer to each gentleMACS C tube using a filter tip and keep the tubes on ice.
11. Transport gentleMACS C tube, Octo Coolers, and snap-frozen tissue to the gentleMACS Octo Dissociator (Figures 2A and 2B).

**CRITICAL:** Transport C tubes and Octo Coolers on wet ice, transport snap-frozen tissue on dry ice.

12. Add snap-frozen tissue to C tube containing lysis buffer. Ensure the lid is screwed on tight. Load the C tube and Octo Coolers onto the gentleMACS Octo Dissociator and then begin nuclei extraction protocol 4C_nuclei_1.

***Note:*** If snap-frozen tissue is in large chunks (>1 cm), try to break up the chunks on dry ice before beginning dissociation.

13. After extraction protocol finishes, remove the C tube containing nuclei in solution and place on ice (Figure 2C).
14. Flow the nuclei suspension through a 70 μm strainer into a 15 mL conical placed on ice (Figure 2D).

***Note:*** To increase nuclei recovery, the C tube can be rinsed with 2 mLs of additional lysis buffer, and the resulting solution can be used to rinse the strainer. Any 70 μm filter can be used, though in our experience strainers with a smaller surface area can clog.

15. Centrifuge the 15 mL conical tube with nuclei at 300 *g* for 5 min at 4°C. Remove supernatant and resuspend in resuspension buffer. Maintain sample on ice (Figure 2E).
16. Filter resuspended nuclei through a 30 μm strainer (Figure 2F) and count nuclei using acridine orange.

**Figure 3:**
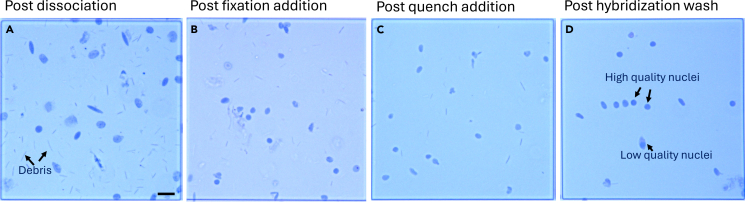
Sequential processing of isolated nuclei (A) Nuclei following dissociation and filtration. (B) Nuclei after formaldehyde fixation added. (C) Nuclei after addition of quenching buffer. (D) Nuclei following post-hybridization washes. Scale bar 25 μm.

**CRITICAL:** To count the nuclei, mix 10 μLs of nuclei sample, 10 μLs of 1×PBS, and 20 μLs of Acridine Orange. Pipette to mix and load volume according to the maximum volume allowed by the fluorescent counter being used. The use of acridine orange is strictly for counting nuclei and should not be used to inform the quality of the isolated nuclei. To check the quality, see note below regarding the use of trypan blue.

***Note:*** It is recommended to fix a maximum of 10 million nuclei using this workflow. If your sample exceeds that amount, either split the sample into 2 tubes to fix or simply move forward with only 10 million nuclei. High nuclei concentration also increase doublet. If it occurs, dilute the sample 1:2**–**1:5 and filter it again through a 30 μm strainer.

***Note:*** It is not necessary to have millions of nuclei to proceed with fixation, though there can be significant losses in nuclei numbers that occur throughout the fixation and filter steps. If planning to multiplex 4 samples and load the maximum number of nuclei into the microfluidic chip, it would be ideal to have at least 200,000 nuclei per sample going into fixation. Users can work with fewer nuclei as input with the expectation that maximal loading of the microfluidic chip might not be possible.

***Note:*** To check the quality of the recovered nuclei, take 5 μL of nuclei solution and mix with 5 μL of 0.04% trypan blue. Load the mixture on to a hemocytometer or disposable hemocytometer chip. Visually inspect the nuclei at 20× or 40× magnification, look for intact, round nuclei with minimal or no blebbing (Figures 3A–3D).

***Note:*** At this stage it is acceptable to have a small number of nuclei doublets or clumping. Prior to loading the fixed and probe hybridized nuclei pool or individual sample, the nuclei are passed through a 30 μm pre-separation filter that effectively removes clumps and doublets. It is most important to have a mostly single nuclei suspension right before loading into the master mix for GEM generation.

### Formaldehyde fixation of isolated nuclei

**Timing: 16–24 h**

17. Pellet the nuclei by centrifuging at 300 *g* for 5 min at 20°C**–**24°C.
18. Resuspend each nuclei sample in 0.5 mL of fixation buffer B.
19. Place samples in the 4°C fridge to fix for 16**–**24 h.

**CRITICAL:** Do not invert or mix the nuclei during fixation incubation.

***Optional:*** The Fixation protocol provides the option to fix 16**–**24 h in the 4°C fridge or to fix for 1 h at 20°C**–**24°C. We have opted to use the 16**–**24 h fixation approach, though we do not anticipate any issues with utilizing the 1 h at 20°C**–**24°C.

***Note:*** Be sure to keep total fixation time consistent between experimental samples that are to be run and analyzed together.

### Quench and probe hybridization

**Timing: 16–24 h**

***Note:*** 10× Genomics provides two demonstrated protocols for probe hybridization and library preparation, depending on whether samples will be mixed into pools or whether they will be standalone samples. To maximize the capability of the kit, we multiplexed our barcoded samples and utilized document CG000787. To process and run individual samples, reference document CG000786.

20. Add 0.5 mL of additive C Pipette mix and centrifuge 850 *g* for 5 min at 20°C**–**24°C.
21. Remove supernatant and resuspend fixed nuclei in 1mL of quenching buffer. Place quenched nuclei on ice.
22. Count fixed nuclei using acridine orange to determine how much of the sample to put into probe hybridization.

***Note:*** To count the nuclei, mix 10 μLs of nuclei sample, 10 μLs of 1×PBS, and 20 μLs of Acridine Orange. Pipette to mix and load volume according to the maximum volume allowed by the fluorescent counter being used. The use of acridine orange is strictly for counting nuclei and should not be used to inform the quality of the isolated nuclei.

***Note:*** Acridine orange dye exhibits lower nonspecific background fluorescence relative to conventional propidium iodide (PI) staining for nuclei or fixed-cell counting.

***Note:*** Users that have sufficient nuclei and intend to pool their samples equally can opt to put the same number of nuclei into probe hybridization for each sample. This approach serves as a pre-hybridization normalization step, allowing users to pool entire volume of sample without needing to perform an additional count.

**Pause point:** If desired, quenched nuclei can be stored at −80°C for up to 6 months with enhancer (10× Genomics) and 50% glycerol. Glycerol should be used after filtering through a 0.2 μm filter.

23. Centrifuge 300,000 fixed, counted cells at 850 × *g* for 5 min at 4°C.

***Note:*** Up to 500,000 nuclei can be used as input into probe hybridization.

24. Remove the supernatant and resuspend each pellet in 40 μLs of Hyb Mix, then transfer to the 8-strip tube.

***Note:*** Keep the sample at 20°C**–**24°C. Do not place it on ice.

***Note:*** Set up probe hybridization, per instructions from 10× Genomics user guide CG000787. In this protocol, probe hybridization was performed in an 8-strip tube format.

25. Add 10 μLs Mouse WTA probes to the 40 μL mixture of Hyb mix and fixed nuclei sample, then gently mix by pipetting.
26. Incubate the samples for hybridization for 16**–**24 h (minimum of 16 h) at 42°C in a thermal cycler with 42°C heated lid, without shaking.

### Post-hybridization pooling and washing

**Timing: 1.5 h**

***Note:*** The following instructions for post hybridization pooling and washing are meant for use when multiplexing 4 samples prior to washing and performing probe hybridization in an 8-strip tube format. Users whose experiment designs require them to deviate from this i.e. multiplexing a different number of samples, washing samples individually, and/or hybridizing in 1.5 mL tubes will need to reference document CG000787 section 2 and follow the specified instructions that apply to their experimental design to ensure proper washing.

27. After 16**–**24 h of incubation, take out the 8-tube strips from the thermal cycler, and add 225 μLs of Post-Hyb Wash Buffer B, pipette mix and count probe hybridized samples using acridine orange.

***Note:*** To count the nuclei, mix 10 μLs of nuclei sample, 10 μLs of 1×PBS, and 20 μLs of Acridine Orange. Pipette to mix and load volume according to the maximum volume allowed by the fluorescent counter being used. The use of acridine orange is strictly for counting nuclei and should not be used to inform the quality of the isolated nuclei.

28. Add 2.1 mLs of Post-Hyb Wash Buffer B into a 5 mL tube.
29. To multiplex 4 samples, pool the 4 samples containing 4 different probe barcodes into the 5 mL tube such that even numbers of nuclei from each sample are represented. Invert the tube 5 times to mix.

***Note:*** If the same number of nuclei were put into probe hybridization for each sample, the entire volume of each sample can be pooled.

30. Centrifuge the tube at 850 × *g* for 5 min at 20°C.
31. Remove the supernatant without disrupting the pellet.

***Note:*** Remove as much supernatant as possible without losing sample. Ideally no more than 30 μLs will be left behind.

32. Add 1 mL of Post-Hyb Wash Buffer B, pipette mix to resuspend the pellet and transfer the entire volume to a 1.5 mL tube.
33. Incubate at 42°C for 10 min in a thermomixer.
34. Centrifuge the 1.5 mL tube at 850 × *g* for 5 min at 20°C.
35. Remove as much supernatant as possible without disrupting the pellet.

**CRITICAL:** Remove as much supernatant as possible without losing sample. Ideally no more than 30 μLs will be left behind.

36. Add 0.5 mLs of Post-Hyb Wash Buffer B. Pipette mix thoroughly.
37. Repeat steps 33**–**36.
38. Incubate at 42°C for 10 min in a thermomixer.
39. Centrifuge the 1.5 mL tube at 850 × *g* for 5 min at 20°C.
40. Remove as much supernatant as possible without disrupting the pellet.

**CRITICAL:** Remove as much supernatant as possible without losing sample. Ideally no more than 30 μLs will be left behind.

41. Resuspend the pellet with 250 μLs of Post-Hyb Resuspension Buffer B and place at 4°C.

***Note:*** This volume is recommended for users attempting to target the maximum cell recovery for the pool. Users targeting less than the maximum recovery can use the calculation of 70% to estimate the number of nuclei retained during the post hybridization washes. This information can inform how much volume of Post-Hyb Resuspension Buffer B is needed to be within the loading range for your experimental design.

***Note:*** Ensure that the resulting sample(s) concentration is in the appropriate range to load the target number of nuclei. Reference the charts near the beginning of reference document CG000787 section 3.1 to determine what nuclei concentrations and volumes are needed to load and recover the desired number of nuclei per GEM reaction based on the number of samples pooled. For example, if 4 samples were multiplexed into a pool, refer to the cell suspension and volume calculator for multiplexing 4 samples to determine concentration range and volumes for input into the master mix. To dilute samples, use post hybridization resuspension buffer and recount with acridine orange. To concentrate samples, spin tube with nuclei at 850 × g for 5 min at 4°C. Remove some volume of supernatant and resuspend nuclei pellet with remaining volume in the tube. Be sure to recount the sample with acridine orange.

42. Flow the pool through a 30 μm pre separation filter and maintain the pool at 4°C.
43. Count nuclei samples or pools using acridine orange to determine the current concentration and whether diluting or concentrating the samples or pools is necessary.

***Note:*** To count the nuclei, mix 10 μLs of nuclei sample, 10 μLs of 1×PBS, and 20 μLs of Acridine Orange. Pipette to mix and load volume according to the maximum volume allowed by the fluorescent counter being used. The use of acridine orange is strictly for counting nuclei and should not be used to inform the quality of the isolated nuclei.

**Pause point:** If desired, washed probe-hybridized nuclei can be stored at −80°C with enhancer (10× Genomics) and 50% glycerol for up to 12 months.

44. Reference the appropriate cell suspension and volume calculator as described in the above note. Use those volumes to input the nuclei sample and post0hyb resuspension buffer B into the GEM master mix and continue to GEM generation according to CG000787.

### FLEX library preparation

**Timing: 2–3 days**

45. Single nuclei RNA-seq libraries are prepared according to the recommendation of the manufacturer (https://www.10xgenomics.com/support/flex-gene-expression/documentation/steps/library-prep/chromium-single-cell-gene-expression-flex-reagent-kits-for-multiplexed-samples).

***Note:*** Assess the TapeStation trace for library quality. User should expect a distinct peak around 260bps.

## Expected outcomes

**Figure 4:**
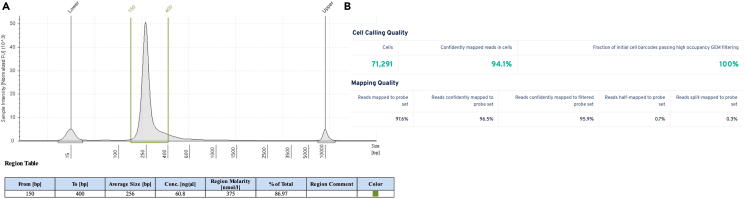
Library quality control (QC) metrics for single nuclei RNA sequencing of mouse colon tissue (A) TapeStation QC result. A distinct single library peak approximately 260 base pairs is expected. (B) Confidently mapped reads in cells should be above 70%. Split-mapped probes should be below 20%.

**Figure 5:**
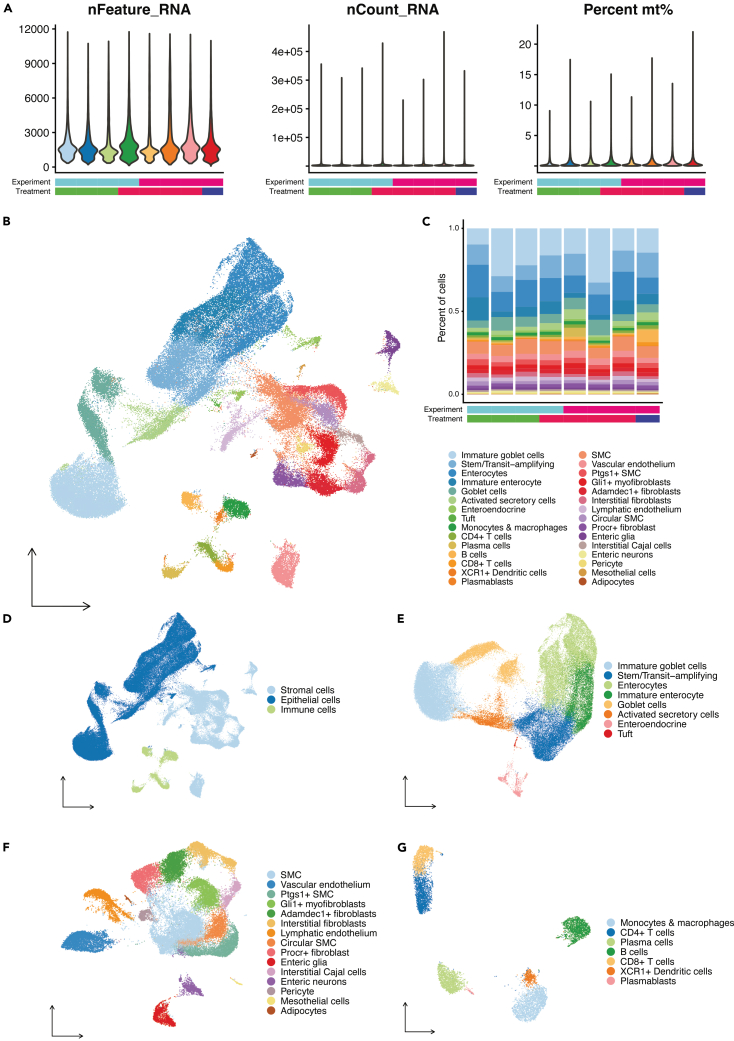
Analyses of mouse colon cell populations by snRNA-seq (A) Quality control violin plots showing the number of detected genes (nFeature_RNA, left), transcripts (nCount_RNA, middle), and the percentage of mitochondrial genes (Percent mt%, right). The experiment batches and treatments for each mouse are indicated by the color bars below. (B) UMAP embedding of all nuclei from the whole colon, colored by detailed cell types. (C) Stacked bar plot showing the composition of each cell type across samples. The experiment batches and treatments for each mouse are annotated by the color bars below. (D) UMAP highlighting the major cell compartments: stromal, epithelial, and immune. (E–G) UMAP embeddings highlighting detailed sub-populations within the (E) epithelial, (F) stromal, and (G) immune compartments.^6^

From 30**–**70 mg of snap-frozen sample, we expect to recover 2**–**6 million nuclei. This protocol is expected to yield a high purity population of intact nuclei with minimal cellular debris (Figure 3). The isolated nuclei should produce high-quality RNA suitable for downstream transcriptomic analyses (Figure 4). Users should expect a distinct, single library peak around 260 base pairs (bps) (Figure 4A). A lower peak averaging at 190bps can be a result of low RNA content, debris, or not enough supernatant removed during the heated washes. Peaks higher than 350bps may be a result of overamplification of the library. User should check that the number of nuclei called by Cell Ranger is reasonably close to the expected number. In our experience, we generally recover at least 80% of what was expected based on the final count of the post hybridized pool or sample. The user should expect that the percent of confidently mapped reads in cells is above 70% (Figure 4B). In our experience, this value is typically greater than 90%. Lower values may indicate excess ambient RNA contamination. The percentage of split-mapped probes should be below 20%. In our experience, this value has been under 2%. An increase in split-mapped probes, i.e., a misligation of probes, could be a result of poor sample quality, low RNA content, or insufficient supernatant removal during wash steps. Before further QC, the number of detected genes (nFeature_RNA), transcripts (nCount_RNA), and percentage of mitochondrial genes (percent mt%) should comparable across experimental batches and treatment groups (Figure 5A). After removing doublets and ambient RNA, major and detailed cell types should be clearly identified and exhibit consistent proportions across batches and treatments (Figures 5B and 5C). The integrated dataset was broadly annotated into major cell compartments (Figure 5D), encompassing the epithelial (Figure 5E), stromal (Figure 5F), and immune (Figure 5G) populations.

When combined with 10× Genomics RNA Flex probe-based sequencing method, which is compatible with formaldehyde fixed samples, the resulting data should enable robust transcriptomic profiling, accurate cell type annotation, and reliable differential gene expression analysis.

## Limitations

This method generates high quality nuclei that can then be formaldehyde fixed and used as input into Fixed RNA profiling, a probe-based assay targeting specific transcript segments across approximately 18,000 protein-coding genes. While this approach enables robust and reproducible cell-type identification, it is inherently limited in capturing transcript features outside the targeted probe regions, including SNPs, alternative splicing events, transcript isoforms, genetic mutations, and long-read transcript information. In addition, because single-nucleus RNA sequencing profiles only nuclear transcripts, it provides reduced representation of cytoplasmic RNA compared to single-cell RNA sequencing. Despite this limitation, snRNA-seq and scRNA-seq are generally comparable in their ability to identify major cell types.^7^ However, for studies focused on rare or highly sensitive immune cell populations, scRNA-seq may be preferable.^6^

Based on the workflow, at least 3 mg of starting material is needed to obtain 300,000 nuclei for the probe hybridization reaction, which may limit use with smaller samples. Another limitation of this protocol is that it requires a commercial dissociator to ensure consistent and reliable results.

Ambient RNA contamination represents a major technical challenge for single-nuclei RNA FLEX sequencing. Although the sequencing quality was overall high in our dataset, we detected ambient RNA contamination primarily originating from highly abundant epithelial and stromal cell populations. To mitigate the issue, we applied SoupX^3^ to estimate and remove ambient RNA contamination. Putative doublets were subsequently detected and removed using DoubletFinder.^4^ Following quality control, we performed batch correction and dimensional reduction using Harmony integrated with Seurat v5.^5^ Unsupervised clustering revealed diverse cell populations across epithelial, immune, and stromal compartments (Figure 5A). We successfully identified most major cell subtypes within each compartment (Figures 5B–5D), consistent with previous published intestinal single-cell atlases.^8^^,^^9^^,^^10^^,^^11^ Moreover, our single-nuclei RNA FLEX sequencing allows samples to be snap-frozen and processed simultaneously, offering a clear advantage for efficient sample handling and experimental design over traditional single-cell sequencing. Notably, our optimized workflow enabled robust detection of several rare cell populations, including interstitial cells of Cajal and enteric nervous system cells (enteric neurons and glia). These populations are particularly challenging to recover using droplet-based scRNA-sequencing because of their fragility during tissue dissociation.^12^^,^^13^^,^^14^

## Troubleshooting

### Problem 1

An additional lower than expected peak is present on the TapeStation trace (Figure 4A).

### Potential solution

Tape Station is required for post-library construction QC. A lower peak averaging at 190bps may result from low RNA content, residual debris, or insufficient removal of supernatant during the post hybridization washes (step-by-step 32**–**38). If possible, go back to non-probe hybridized fixed nuclei and proceed through probe hybridization to library preparation, being sure to remove as much supernatant as possible during the post hybridization washes.

### Problem 2

Debris in the sample.

### Potential solution

This issue may arise when tissue fragments and cell membranes are not adequately removed during filtration, leading to inaccurate nuclei counts and poor data quality. To mitigate this, repeat filtration step using a 30 μm pore-size filter and carefully verify that all sequential steps are performed correctly. Notably, fixing the tissue prior to dissociation can increase cellular debris. Therefore, snap-frozen samples should be dissociated first, followed by fixation of isolated nuclei.

### Problem 3

Ambient RNA contamination.

### Potential solution

Adding an additional post-hybridization washing step may help reduce this contamination. Additionally, Computational approaches can be used to remove background noise. For example, SoupX can effectively correct for ambient RNA contamination. In mouse colon samples, expression of the *Mut2* gene serves as a useful indicator for identifying and removing ambient RNA.

### Problem 4

Poor performance in Cell Ranger quality control metrics.

### Potential solution

Ensure that sample preparation is performed as described, particularly the step-by-steps 6**–**8 and 10**–**18. This protocol is specifically optimized for snap-frozen colon tissue and follows a dissociation-then-fixation workflow. In colon studies, mucosal scraping is considered a way to reduce contamination from muscular layers in colon tissue. However, this approach can introduce mechanical damage to nuclei, resulting in suboptimal data quality and is not recommended.

### Problem 5

Lower number of nuclei recovered from the Miltenyi nuclei isolation protocol than expected.

### Potential solution

A lower-than-expected number of nuclei recovered after dissociation could be a result of inefficient dissociation that can be helped by breaking up bigger chunks of tissue before placing the sample into the C tube and initiating dissociation.

## Resource availability

### Lead contact

Further information and requests for resources and reagents should be directed to and will be fulfilled by the lead contact, Dr. Ming Yu (myu@fredhutch.org).

### Technical contact

Technical questions regarding this protocol should be directed to the designated technical contacts, Jin-Hee Kim (jkim6@fredhutch.org) and Annalyssa N Long (along@fredhutch.org) who will provide responses.

### Materials availability

No new materials were generated in this study.

### Data and code availability

The code generated during this study is available in the GitHub repository at https://github.com/Yumo-Xie/snRNA-seq_Protocol.

## Acknowledgments

We thank Daniel Hunter from 10× Genomics and Nathan Dunaway from Miltenyi Biotec for their helpful discussions and valuable input during the conceptualization of this protocol.

This work was supported by grants U01AG077920 (M.Y.), U2CCA271902 (M.Y.), U54CA274374 (M.Y.), and R50CA233042 (M.Y.). M.Y. is also supported by the 10.13039/100018138Kuni Foundation, Cottrell Family Fund. Institutional support is provided by the 10.13039/100000002NIH P30 CA015704 of the 10.13039/100005895Fred Hutch/10.13039/100007812University of Washington/10.13039/100010514Seattle Children’s Cancer Consortium, which includes the Genomics & Bioinformatics Shared Resource (RRID:SCR_022606).

## Author contributions

J-H.K. and A.N.L. conceptualized the method, performed the experiments, and wrote the original manuscript. Y.X. performed the bioinformatic analysis. K.T.C. and S.R.N-T. performed the experiments. M.Y. and A.E.E. supervised and reviewed the manuscript.

## Declaration of interests

The authors declare no competing interests.
